# Supplementary material for: Proteomics, Phosphoproteomics and Mirna Analysis of Circulating Extracellular Vesicles through Automated and High-Throughput Isolation
Source: Cells. 2022 Jun 29;11(13):2070. doi: 10.3390/cells11132070 (PMC9265938; doi:10.3390/cells11132070)
Supplement: Supplementary file 1 [file cells-11-02070-s001.zip › cells-1731119-supplementary/cells-1731119-supplementary figures.pdf]

Proteomics, phosphoproteomics and miRNA analysis of circulating extracellular vesicles through automated and high-throughput isolation

Hao Zhang[1,2#], Yu-han Cai[1#], Yajie Ding[1], Guiyuan Zhang[1,3], Yufeng Liu[1,3], Jie Sun[1], Dong Wei[1], Yuchen Yang[4], Zhen Zhan[1], Anton Iliuk[5], Zhongze Gu[1], Yanhong Gu[4], W. Andy Tao[5,6*]

Supplementary Material

SUPPLEMENTARY FIGURE CAPTION

Figure S1 Optimization of automatic extraction instrument. A. the magnetic beads dosage. B. Incubation time. C. Washing time. D. Elution speed.

Figure S2 RPS (Range 150 nm-500 nm) characterization of EVs isolated by the three methods.

Figure S3 Intersection of exosome proteomics results with different capture methods and TOP100 protein in ExoCarta database

Figure S4 Workflow of EV miRNA analyses of urine samples from patients with prostatitis or prostatosis (Control) and prostate cancer.

Figure S5 PCA (Principal Components Analysis) of EV protein (Left) and phosphorylated protein (Right).

Figure S6 GO analysis of EV differential proteins.

Figure S7 KEGG analysis of EV differential proteins.

Figure S8 Other potential biomarkers of urinary exosomes in prostate cancer

Figure S9 Comparison of miR-125b/miR-145 results in urine exosomes between control and prostate cancer groups

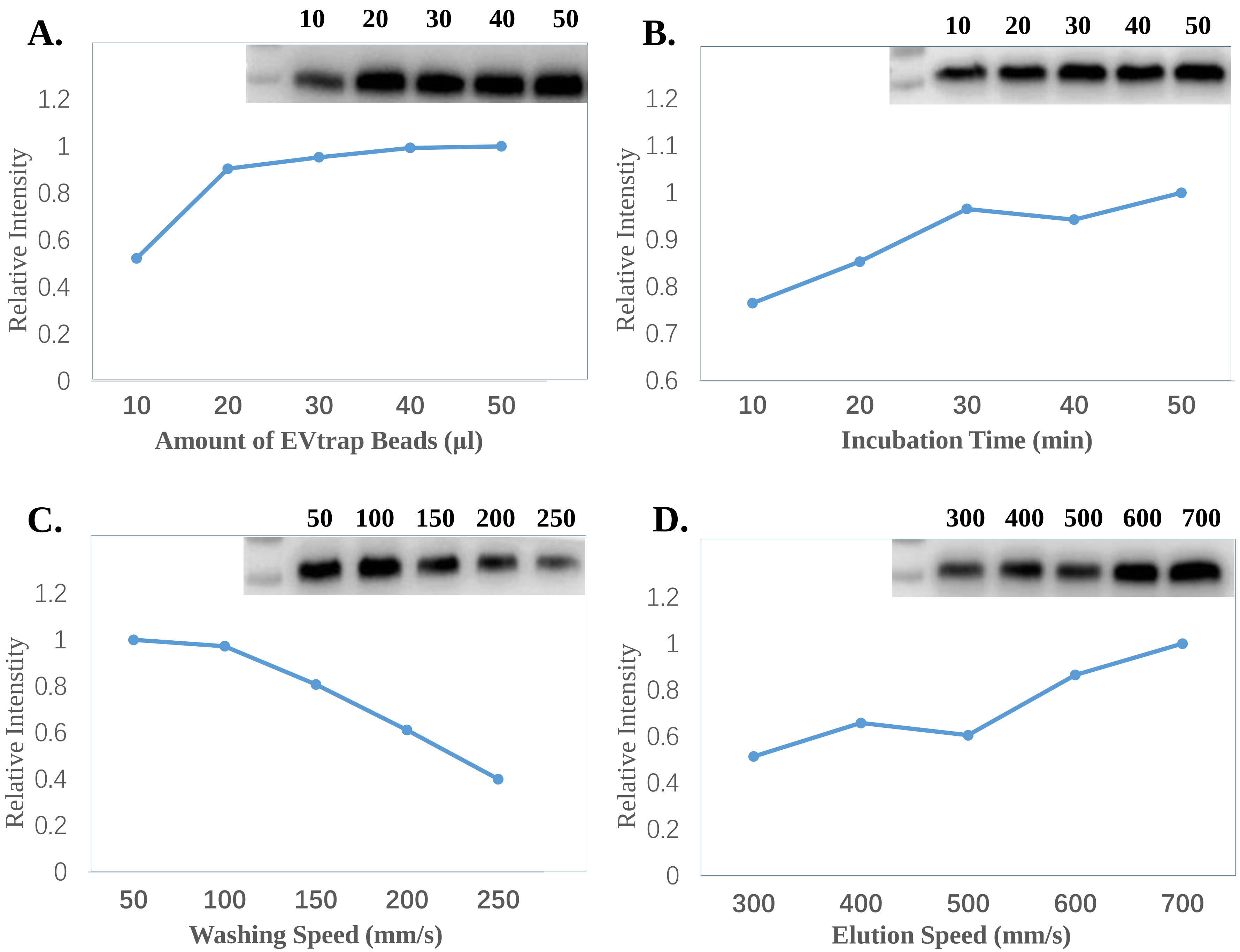

A.
10 20 30 40 50
Relative Intensity
0 0.2 0.4 0.6 0.8 1 1.2
10 20 30 40 50
Amount of EVtrap Beads (μl)
B.
10 20 30 40 50
Relative Intensiy
0.6 0.7 0.8 0.9 1 1.1 1.2
10 20 30 40 50
Incubation Time (min)
C.
50 100 150 200 250
Relative Intenstity
0 0.2 0.4 0.6 0.8 1 1.2
50 100 150 200 250
Washing Speed (mm/s)
D.
300 400 500 600 700
Relative Intensity
0 0.2 0.4 0.6 0.8 1 1.2
300 400 500 600 700
Elution Speed (mm/s)

Figure S1

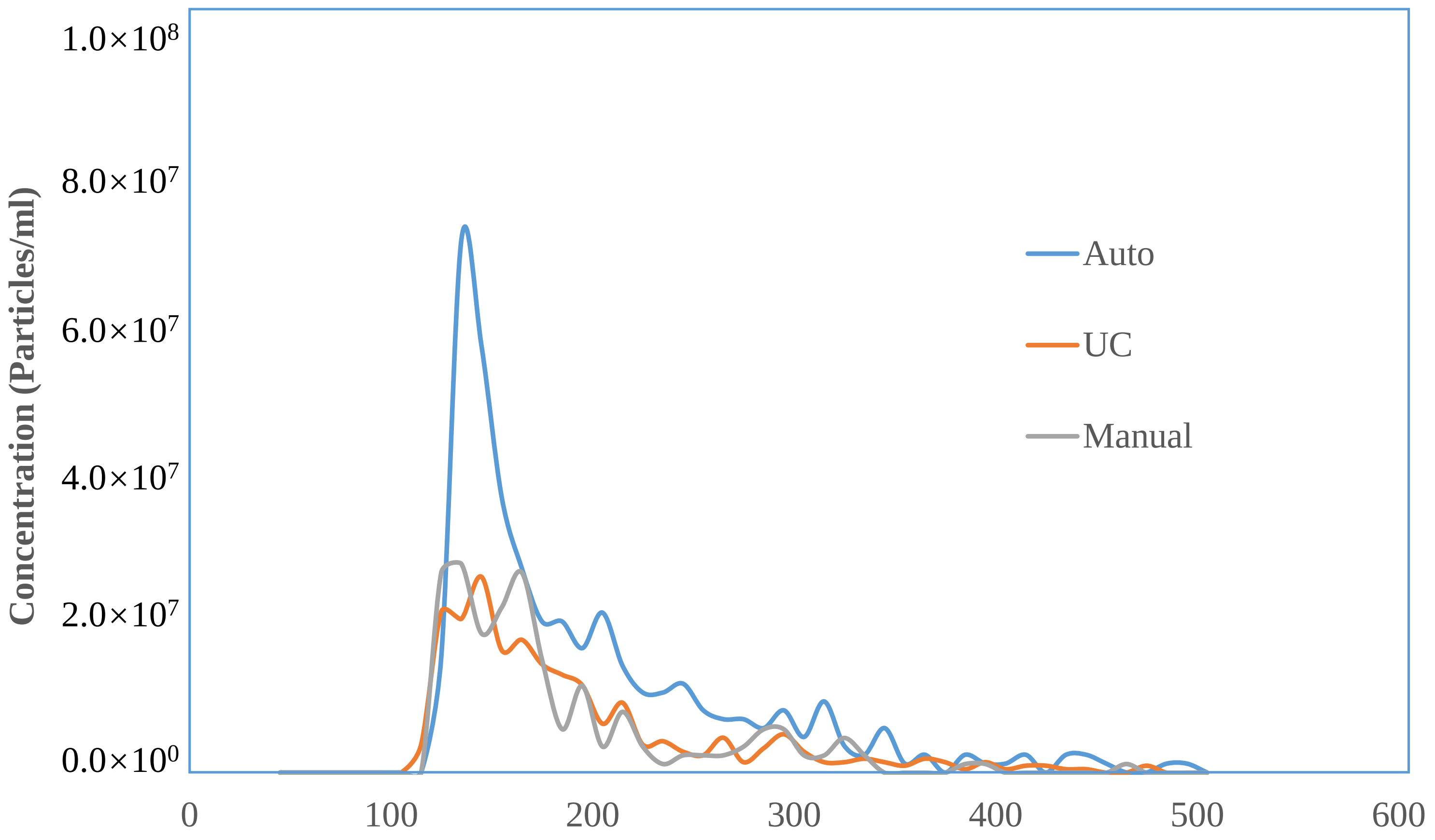

Concentration (Particles/ml)
1.0×10^8
8.0×10^7
6.0×10^7
4.0×10^7
2.0×10^7
0.0×10^0
0
100
200
300
400
500
600
Auto
UC
Manual

Figure S2

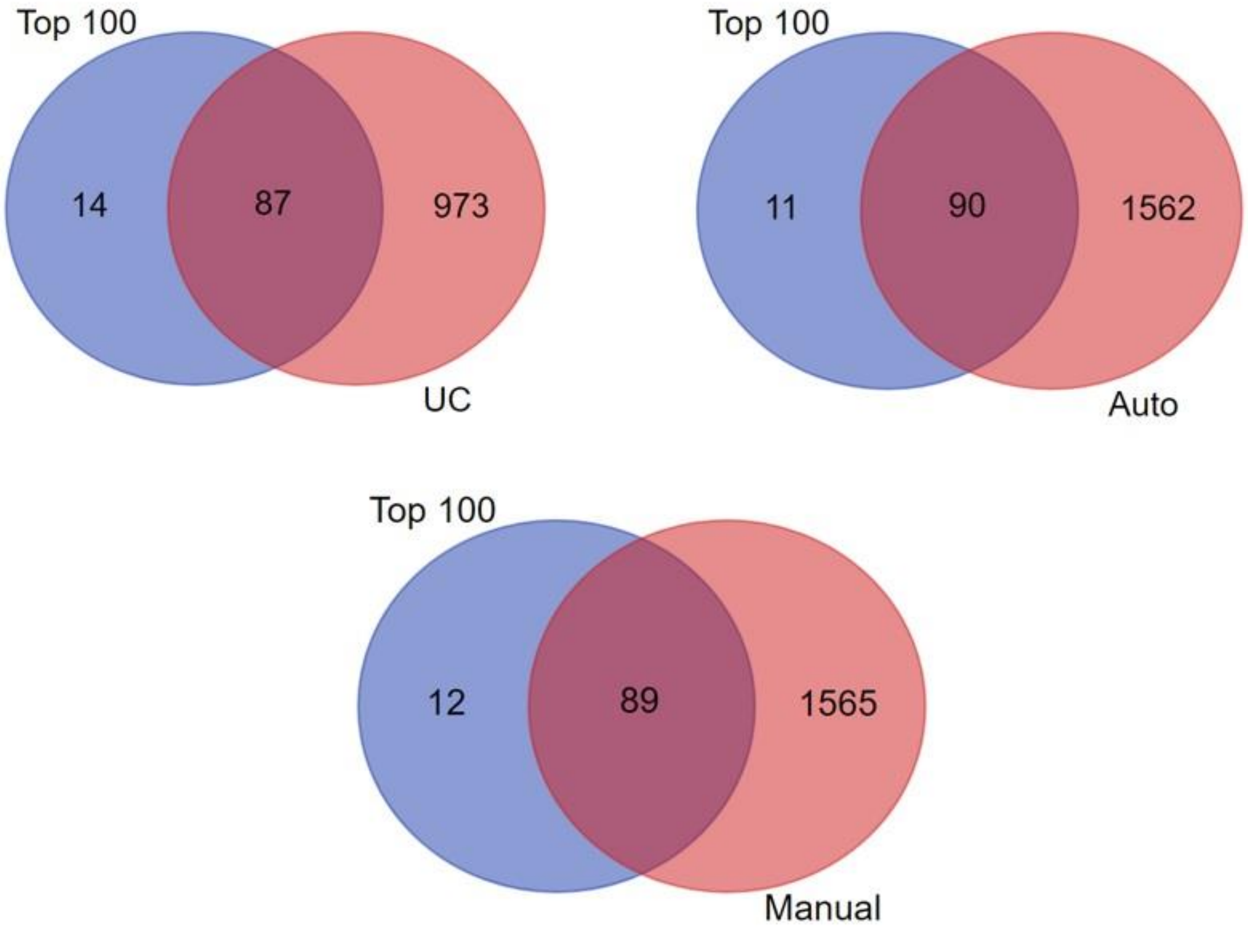
Top 100
14
87
973
UC
Top 100
11
90
1562
Auto
Top 100
12
89
1565
Manual

Figure S3

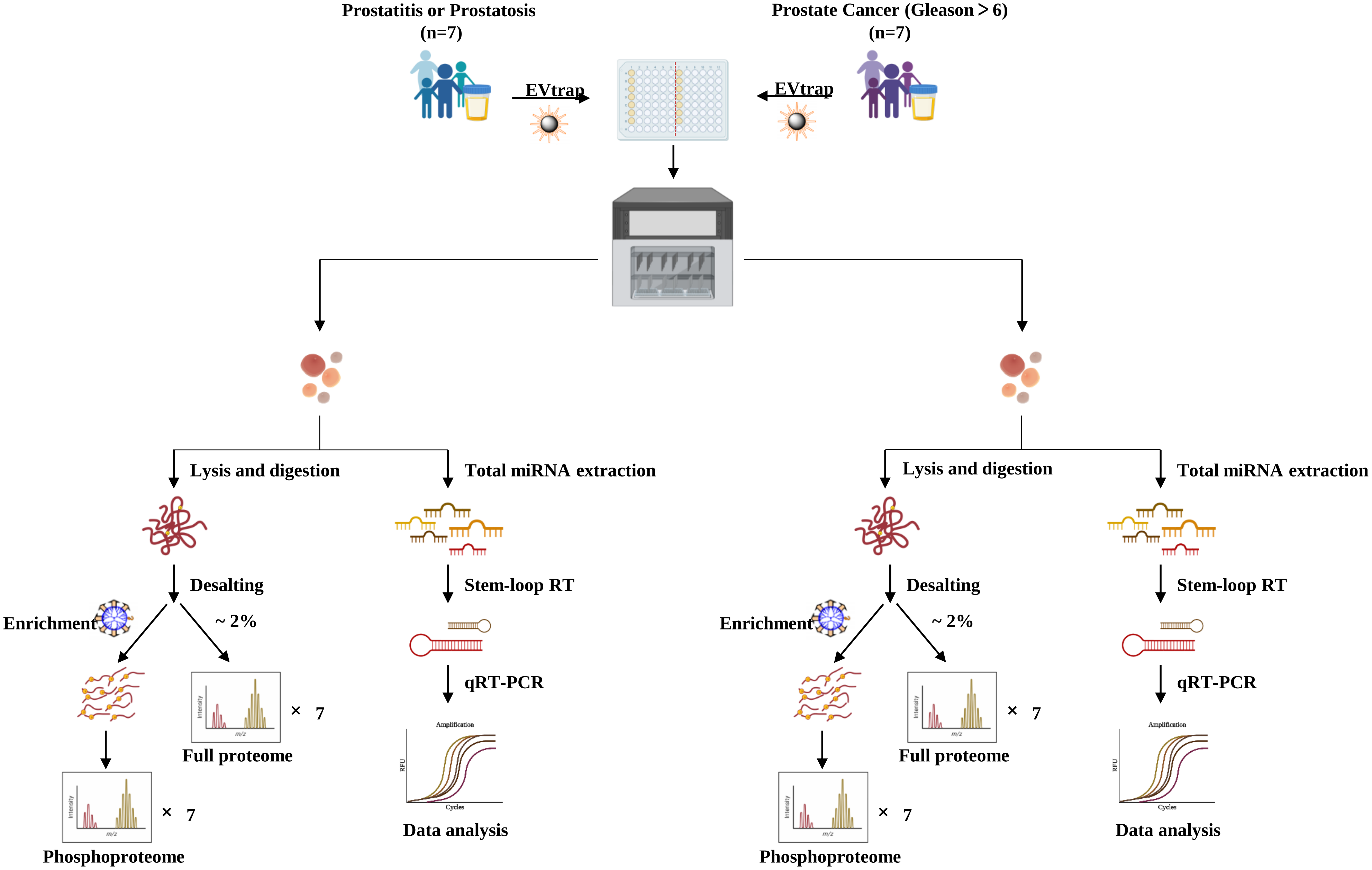
Prostatitis or Prostatosis
(n=7)
Prostate Cancer (Gleason＞6)
(n=7)
EVtrap
EVtrap
Lysis and digestion
Total miRNA extraction
Lysis and digestion
Total miRNA extraction
Desalting
Stem-loop RT
Desalting
Stem-loop RT
Enrichment
~ 2%
Enrichment
~ 2%
× 7
Full proteome
qRT-PCR
× 7
Full proteome
qRT-PCR
× 7
Phosphoproteome
Data analysis
× 7
Phosphoproteome
Data analysis

Figure S4

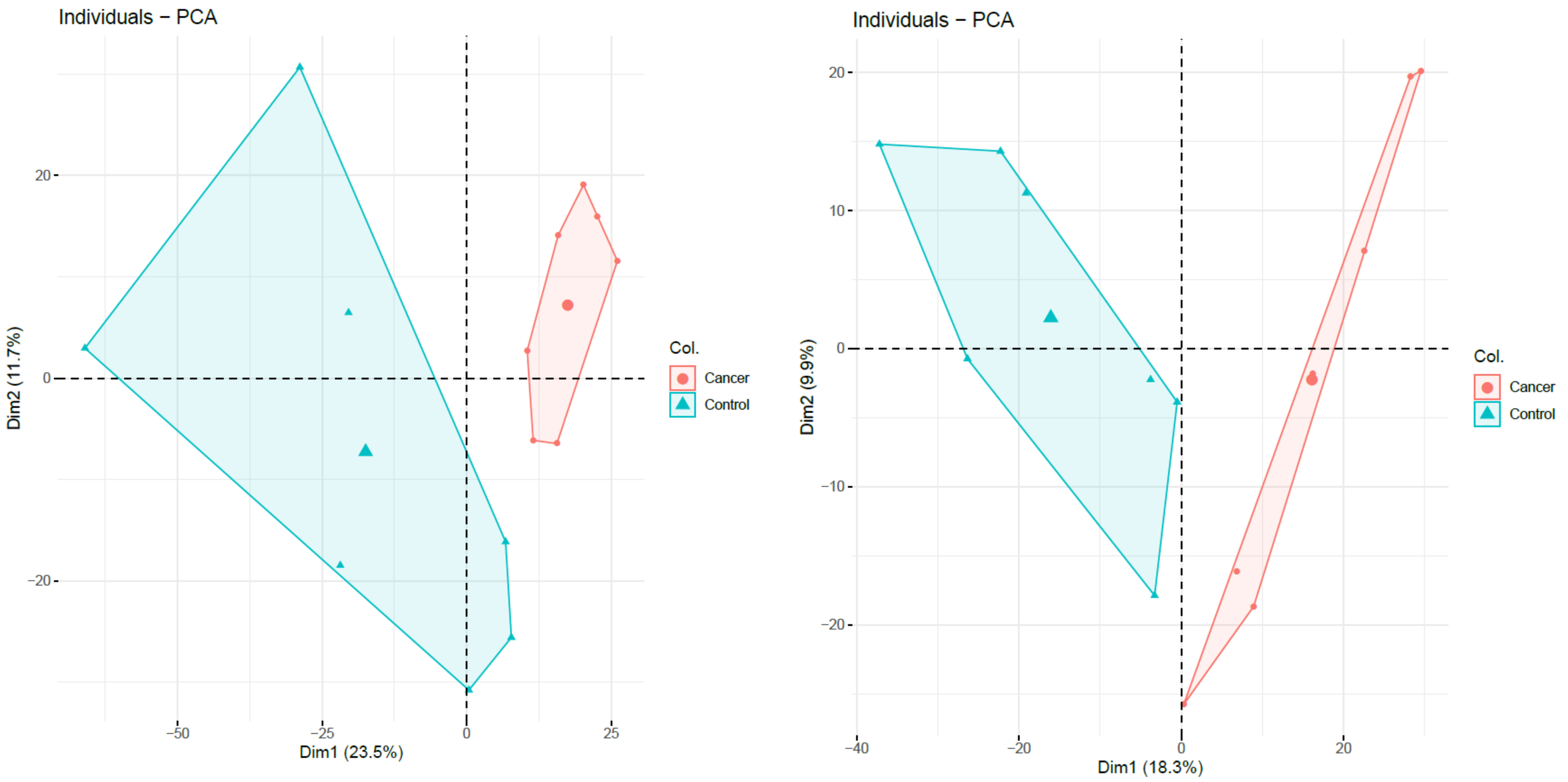

Individuals – PCA
Dim2 (11.7%)
Dim1 (23.5%)
20
0
–20
–50
–25
0
25
Col.
Cancer
Control
Individuals – PCA
Dim2 (9.9%)
Dim1 (18.3%)
20
10
0
–10
–20
–40
–20
0
20
Col.
Cancer
Control

Figure S5

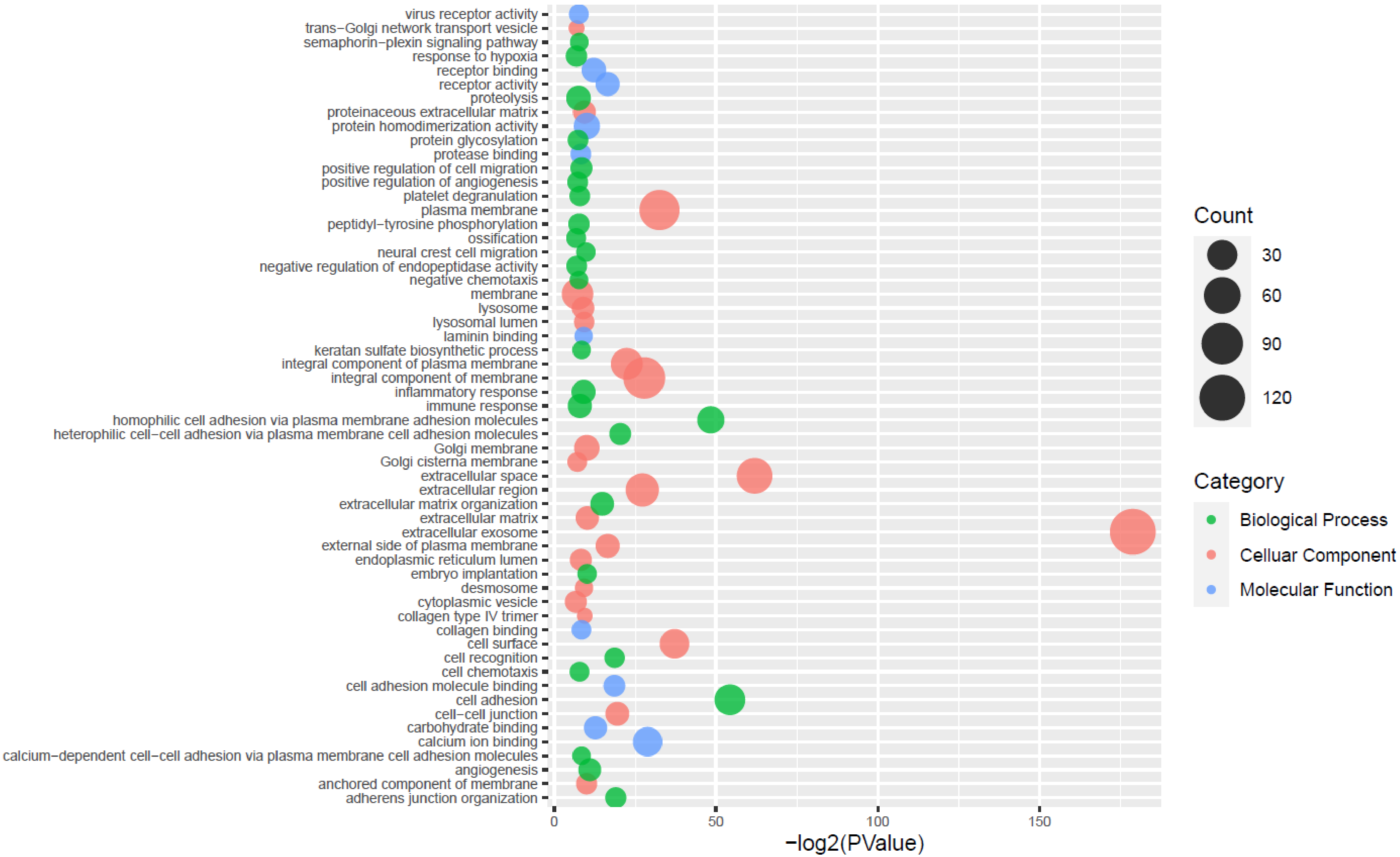
virus receptor activity
trans−Golgi network transport vesicle
semaphorin−plexin signaling pathway
response to hypoxia
receptor binding
receptor activity
proteolysis
proteinaceous extracellular matrix
protein homodimerization activity
protein glycosylation
protease binding
positive regulation of cell migration
positive regulation of angiogenesis
platelet degranulation
plasma membrane
peptidyl−tyrosine phosphorylation
ossification
neural crest cell migration
negative regulation of endopeptidase activity
negative chemotaxis
membrane
lysosome
lysosomal lumen
laminin binding
keratan sulfate biosynthetic process
integral component of plasma membrane
integral component of membrane
inflammatory response
immune response
homophilic cell adhesion via plasma membrane adhesion molecules
heterophilic cell−cell adhesion via plasma membrane cell adhesion molecules
Golgi membrane
Golgi cisterna membrane
extracellular space
extracellular region
extracellular matrix organization
extracellular matrix
extracellular exosome
external side of plasma membrane
endoplasmic reticulum lumen
embryo implantation
desmosome
cytoplasmic vesicle
collagen type IV trimer
collagen binding
cell surface
cell recognition
cell chemotaxis
cell adhesion molecule binding
cell adhesion
cell−cell junction
carbohydrate binding
calcium ion binding
calcium−dependent cell−cell adhesion via plasma membrane cell adhesion molecules
angiogenesis
anchored component of membrane
adherens junction organization
0
50
100
150
−log2(PValue)
Count
30
60
90
120
Category
Biological Process
Celluar Component
Molecular Function

Figure S6

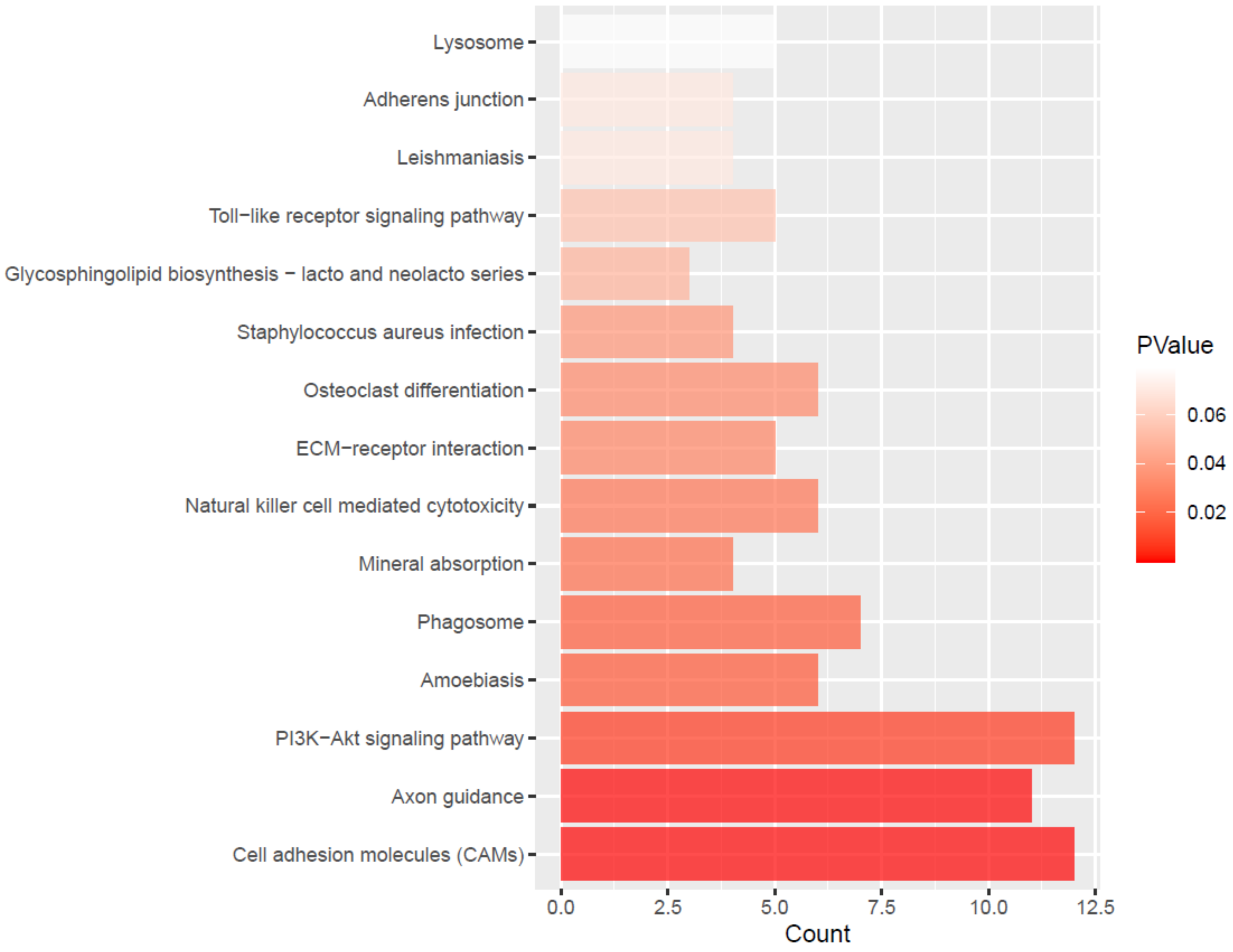
Lysosome
Adherens junction
Leishmaniasis
Toll−like receptor signaling pathway
Glycosphingolipid biosynthesis − lacto and neolacto series
Staphylococcus aureus infection
Osteoclast differentiation
ECM−receptor interaction
Natural killer cell mediated cytotoxicity
Mineral absorption
Phagosome
Amoebiasis
PI3K−Akt signaling pathway
Axon guidance
Cell adhesion molecules (CAMs)
0.0
2.5
5.0
7.5
10.0
12.5
Count
PValue
0.06
0.04
0.02

Figure S7

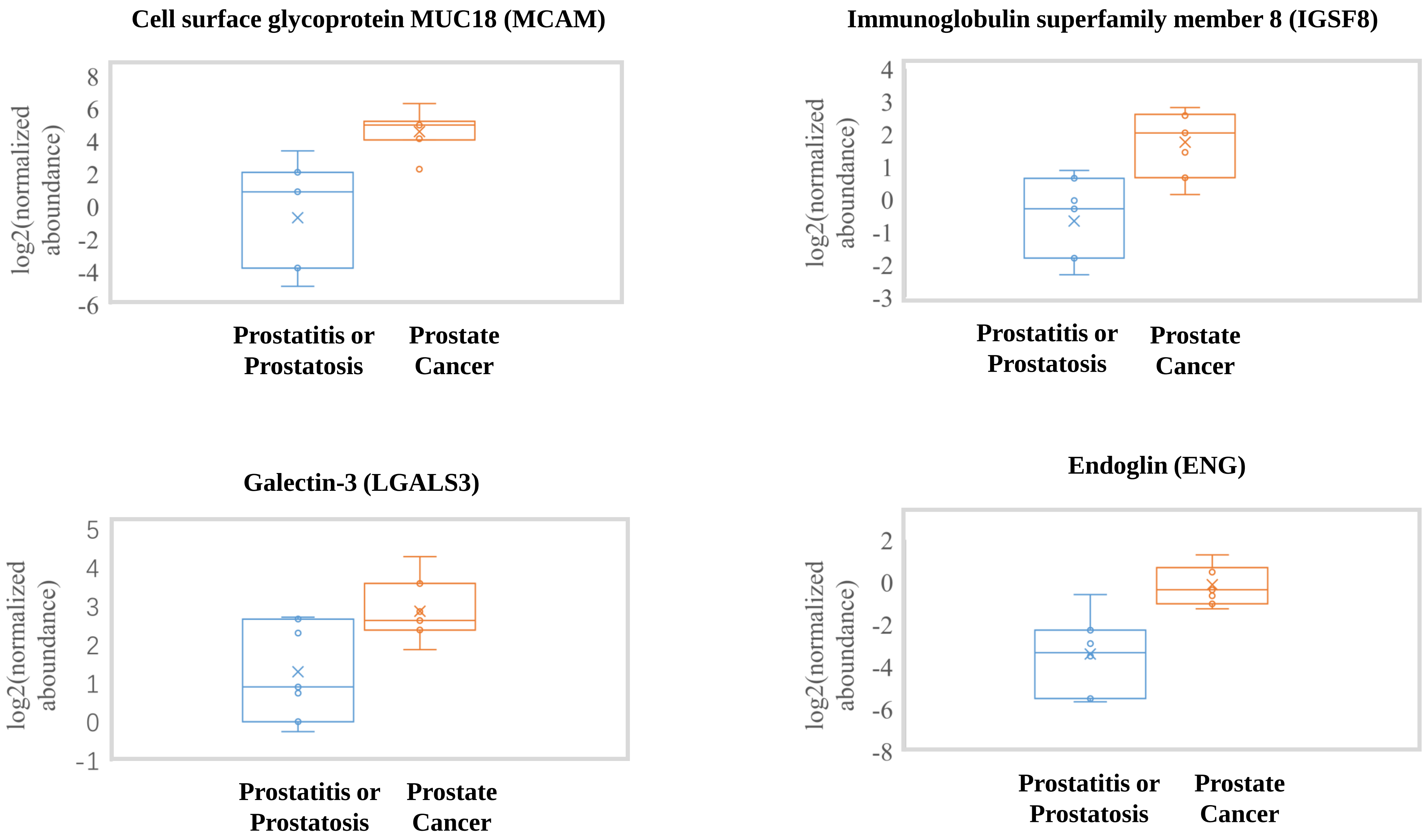
Cell surface glycoprotein MUC18 (MCAM)
log2(normalized aboundance)
8
6
4
2
0
-2
-4
-6
Prostatitis or Prostatosis
Prostate Cancer
Immunoglobulin superfamily member 8 (IGSF8)
log2(normalized aboundance)
4
3
2
1
0
-1
-2
-3
Prostatitis or Prostatosis
Prostate Cancer
Galectin-3 (LGALS3)
log2(normalized aboundance)
5
4
3
2
1
0
-1
Prostatitis or Prostatosis
Prostate Cancer
Endoglin (ENG)
log2(normalized aboundance)
2
0
-2
-4
-6
-8
Prostatitis or Prostatosis
Prostate Cancer

Figure S8

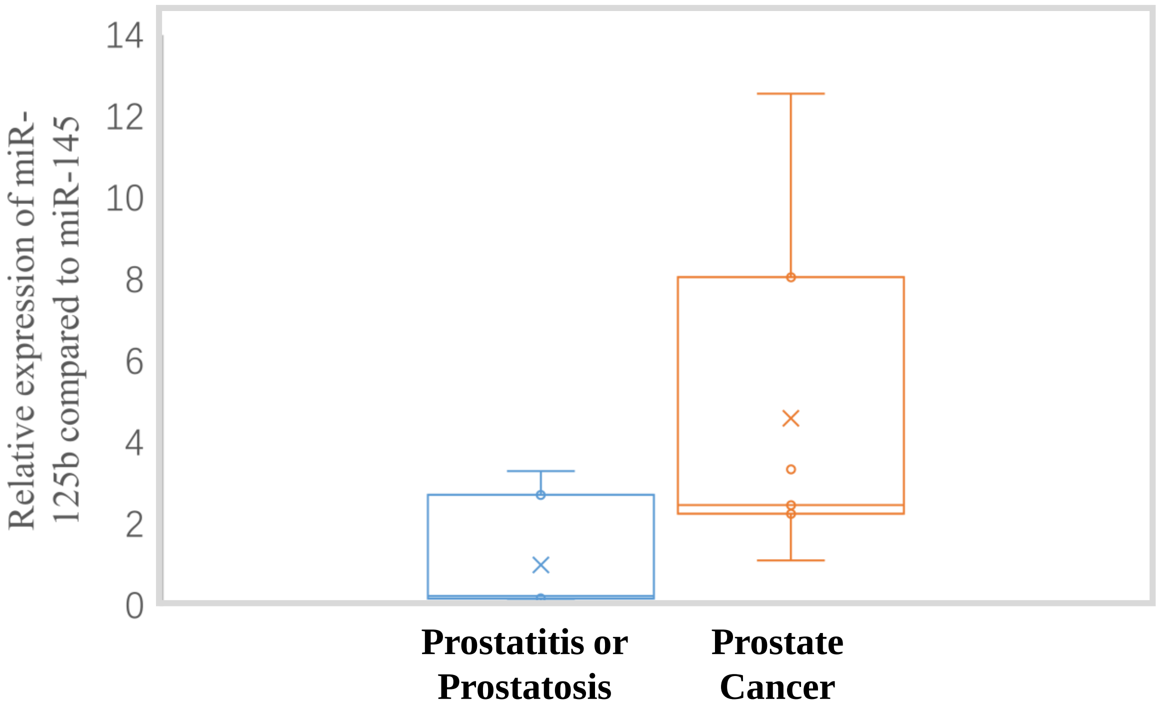
Relative expression of miR-125b compared to miR-145
14
12
10
8
6
4
2
0
Prostatitis or Prostatosis
Prostate Cancer

Figure S9
